# SSeCKS/Akap12 suppresses metastatic melanoma lung colonization by attenuating Src-mediated pre-metastatic niche crosstalk

**DOI:** 10.18632/oncotarget.26067

**Published:** 2018-09-11

**Authors:** Masashi Muramatsu, Shin Akakura, Lingqiu Gao, Jennifer Peresie, Benjamin Balderman, Irwin H. Gelman

**Affiliations:** ^1^ Institute of Resource Development and Analysis, Kumamoto University, Kumamoto 860-0811, Japan; ^2^ Frontiers in Bioscience Research Institute in Aging and Cancer, Irvine 92618, CA, USA; ^3^ Department of Cancer Genetics and Genomics, Roswell Park Comprehensive Cancer Center, Buffalo 14263, NY, USA

**Keywords:** SSeCKS/Akap12, pre-metastatic niche, melanoma, lung endothelial cells, adhesion

## Abstract

SSeCKS/Gravin/AKAP12 (SSeCKS) controls metastasis-associated PKC and Src signaling through direct scaffolding activity. SSeCKS is downregulated in the metastases of many human cancer types, and its forced re-expression suppresses the metastatic behavior of prostate cancer cells. SSeCKS is also downregulated in breast and prostate cancer stroma, and SSeCKS-null mice (KO) are metastasis-prone, suggesting a role in suppressing formation of the pre-metastatic niche. Here, we show that lung colonization and metastasis formation by B16F10 and SM1WT1[*Braf*^V600E^] mouse melanoma cells is 9-fold higher in syngeneic KO compared to WT hosts, although there is no difference in orthotopic tumor volumes. Although melanoma cells adhered equally to KO or WT lung fibroblasts (LF), co-injection of melanoma cells with KO (vs. WT) LF increased lung macrometastasis formation in WT hosts, marked by increased melanoma colonization at foci of leaky vasculature. Increased melanoma adhesion on KO lung endothelial cells (LEC) was facilitated by increased E-Selectin levels and by increased STAT3-regulated secretion of senescence-associated factors from KO-LF, such as Vegf. Finally, the ability of SSeCKS to attenuate IFNα-induced Stat3 activation in KO-LF required its Src-scaffolding domain. Taken together, these data suggest that SSeCKS normally suppresses metastatic colonization in the lung by attenuating the expression of Selectin adhesion proteins, which can be controlled autonomously by local endothelial cells or enhanced by senescence factors secreted by neighboring fibroblasts in a SSeCKS-regulated, Src/Stat3-dependent manner.

## INTRODUCTION

The vast majority of cancer-related deaths result from metastatic outgrowths at distal sites, where the combined selection for tumor cells with increased chemotactic and invasive abilities, and the ability to adapt to new microenvironments, correlates with increased resistance to therapies that are efficacious against primary-site tumors [1]. There is growing appreciation that metastasis is controlled by a crosstalk between secreted factors produced by primary-site tumor cells and cells composing pre-metastatic niches (PMN) at distal sites [2–4]. Tumor cells “educate” PMN cells by secreting soluble factors such as S100A8 and S100A9 [5], as well as regulatory miRNAs and proteins packaged in exosomes and vesicles [6], which facilitate the recruitment of Mac1^+^ myeloid [5] and VEGFR1^+^ bone marrow-derived hematopoietic cells [7] to lung PMN. In response, sensitized PMN cells increase surface expression of fibronectin, which increases colonization of myeloid and bone marrow cells via their upregulation of fibronectin-binding integrin-α4β1, and increase secretion of chemokines such as SDF-1(CXCL12), TNF-α, TGF-β, VEGF-A, CXCL12 or PLGF [2, 8] that serve as tumor attractants. Metastatic colonization is subsequently facilitated by an increased expression by local endothelial cells of P- or E-Selectin [9–11], ICAM-1 [12] or VCAM-1 [13]. For example, B16F10 melanoma cells ectopically expressing the P-/E-Selectin ligand, sialyl Lewis X, exhibit higher frequencies of lung metastases from orthotopic primary sites [14], whereas orthotopic tumors grown in E-Selectin-null mice showed decreased lung metastasis formation compared to WT hosts [15].

An area still poorly understood concerns the signaling pathways and conditions that control the expression of adhesion proteins, such as Selectins, on endothelial cells that facilitate metastatic colonization. STAT3 signaling in endothelial cells, likely activated downstream of JAK or Src-family kinases [16], was shown to be critical for metastastic but not primary-site growth by murine Lewis lung carcinoma cells, correlating with endothelial cell upregulation of P-/E-Selectins and tumor cell adhesion [17]. PMN exhibit increased markers of inflammation, many of which typify those secreted by senescent stromal cells [4], and indeed, senescent fibroblasts are known to promote the ability of poorly metastatic cells to form lung metastases in experimental models [18].

SSeCKS/Gravin/AKAP12 (“SSeCKS”) functions as a metastasis suppressor through its ability to scaffold multiple mediators of oncogenic signaling, including PKC, PKA, Src-family kinases (SFK), phosphoinositol phosphates, F-actin and cyclins [19]. SSeCKS/*AKAP12* expression is downregulated in many human cancers compared to normal, untransformed tissues, but its greatest relative losses are found in metastatic lesions, where its transcriptional downregulation and/or gene deletion correlate with metastatic progression in colon, gastric, esophageal and prostate cancer in a tumor cell autonomous manner [19]. For example, the time-to-onset of castration-recurrent prostate cancer correlating with *AKAP12* loss is roughly three times less than cases without *AKAP12* loss [20].

Direct experimental data bolster the notion that SSeCKS expression in tumor cells suppresses metastatic progression. For example, SSeCKS re-expression in MAT-LyLu prostate cancer cells suppresses the formation of macroscopic lung metastases spontaneously arising from subcutaneous or orthotopic sites, without affecting primary-site tumor growth [21]. Transgenic mice lacking *Akap12* and prostate-specific *Rb* exhibit high levels of metastatic dissemination to local lymph nodes of cells derived from prostatic intraepithelial neoplasias [20].

SSeCKS/AKAP12 expression in microenvironmental cells (non-tumor cell autonomous) also controls metastatic progression. Loss of AKAP12 expression is noted in the tumor stroma in breast and prostate cancer, compared to non-tumor-associated stroma [22]. SSeCKS-null mice form increased numbers of carcinogen (DMBA/TPA)-induced tumors and metastases [23], correlating with upregulated levels of FAK, a known promoter of skin cancer in this model [24, 25]. Moreover, injection of murine B16F10 (*Braf*^WT^) or SM1WT1 (*Braf*^600E^) melanoma cells induced increased numbers of peritoneal metastases in syngeneic KO vs. WT hosts, although there was no difference in the growth of primary-site intradermal tumors [22]. The increased metastasis in KO hosts was based on Cxcr3-dependent tumor cell chemotaxis attracted to an increased expression and secretion of Cxcl9/10 from peritoneal membrane myofibroblasts.

Here, we followed up on the finding that injection of B16F10 or SM1WT1 into SSeCKS-null mice produce ∼9-fold higher numbers of lung macrometastases than in WT hosts. Our data indicate that the enhanced lung metastasis is mediated by an increase in tumor cell adhesion to SSeCKS-null lung endothelial cells and by an increased secretion of soluble factors by SSeCKS-null lung fibroblasts that upregulate E- and P-Selectins, known tumor cell adhesion factors [26], on lung endothelial cells. Our data strongly suggest that SSeCKS expression in several microenvironmental cell types controls metastatic melanoma progression in the lung through secreted crosstalk factors and through increased colonization mechanisms.

## RESULTS

**Figure 1:**
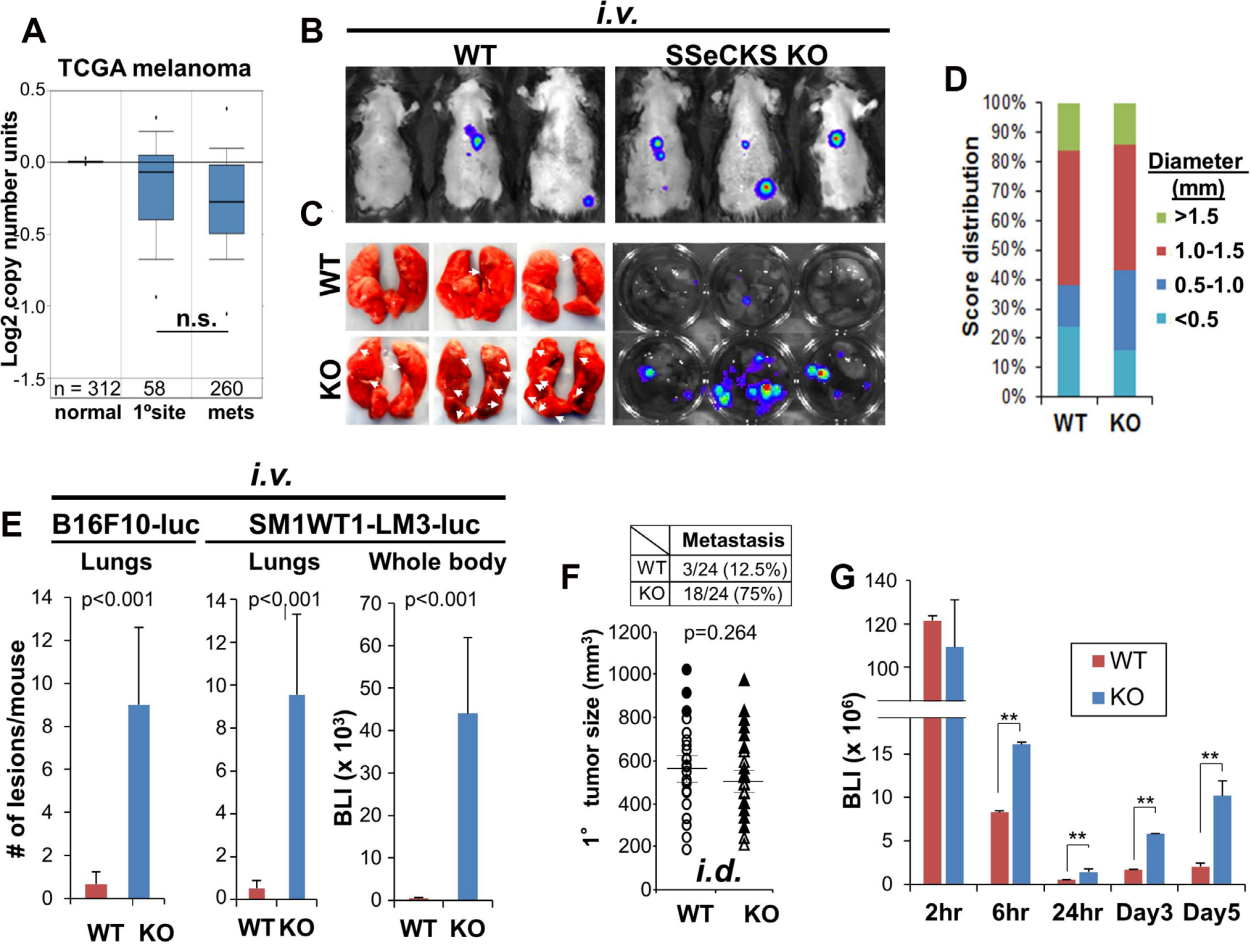
Loss of SSeCKS in the microenvironment promotes metastasis through increased cancer cell attachment in the lung (**A**) TCGA melanoma data showing relative *AKAP12* expression levels in normal prostates, primary prostate cancers and metastases (n, # of cases/group). n.s., not significant (*p* > 0.08). (**B**) Representative lung IVIS images of WT or KO mice injected *i.v.* with B16F10-luc. (**C**) *Left panel.* Fixed lungs from injected WT or KO hosts. Arrows, macrometastases. *Right panel.* Lungs incubated in D-Luciferin solution for 10 min, then imaged by IVIS. (**D**) Size distribution and relative frequency of macrometastases (diameter, in mm) in lungs of WT or KO mice 14 d after *i.v.* B16F10-luc injection. *n =* 15. (**E**) Number of metastatic lesions/mouse or BLI (whole body) in WT or KO hosts after *i.v.* injection of B16F10-luc or SM1WT1-LM3-luc. *n =* 12 mice/group, Error bars, SEM. (**F**) *Bottom.* Volumes of primary *i.d.* B16F10-luc tumors in WT or KO hosts (*n =* 24/group). Filled circles (WT mice) and filled triangles (KO mice) indicate having metastasis. *Top.* Number and percentage of mice with lung macrometastases. (**G**) BLI of lungs at the time points after B16F10-luc *i.v.* injection. *n =* 6/time point. Error bars indicate SEM. ^**^p < 0.001.

Our previous data identified roles for SSeCKS expressed by tumor cells [19] and by microenvironmental cells [23, 22] in metastasis suppression. Melanoma is ideal to study non-tumor cell-autonomous metastasis-suppressor roles for SSeCKS because, unlike many other cancers [19], SSeCKS/*AKAP12* expression is not super-downregulated in human melanoma metastases compared to primary-site lesions (Figure 1A), and the loss of *AKAP12* expression does not alter melanoma survival (Supplementary Figure 1A). Although data for melanoma-associated stroma are not available, stroma isolated from prostate cancers exhibit decreased AKAP12 expression compared to that in normal or hyperplastic stroma [22] (Supplementary Figure 1B), and this does not correlate with increased tumor inflammation (Supplementary Figure 1C). However, the tail-vein injection of B16F10 (*Braf*^WT^) murine melanoma cells into syngeneic SSeCKS-null (KO) mice produced increased metastatic burden (Figure 1B) and ∼9-fold more macroscopic lung metastases (Figure 1C and 1E) compared to similar injections in WT mice. Similar results were produced using SM1WT1-LM3-luc (Figure 1E), a *Braf*^C600E^ melanoma line with increased metastatic potential [22], indicating that the enhanced macro-metastasis in KO hosts was likely *Braf*-independent. In contrast, orthotopic (*i.d.*) B16F10 tumors grew to similar sizes in KO and WT hosts (Figure 1D). Moreover, whereas the largest primary-site tumors in WT mice correlated with the highest metastatic potential, metastatic potential in KO mice showed no such correlation (Figure 1F). These data are consistent with a non-tumor cell autonomous role for SSeCKS in metastasis suppression in this model.

Among the temporal events that define the metastatic cascade in experimental metastasis models, colonization at peripheral sites is one of the first to influence metastatic potential. To test this, WT or KO mice were injected *i.v.* via tail-veins and then cohorts were imaged for luciferase marker activity in host lungs over the first hours and days. This analysis showed 2- to 5-fold increased colonization of B16F10 in KO (vs. WT) lungs starting at 6h and sustained until 5d post-injection (Figure 1G). This strongly suggests that the loss of SSeCKS in lung cells increases melanoma cell adhesion, resulting in increased colonization of disseminating cells.

**Figure 2:**
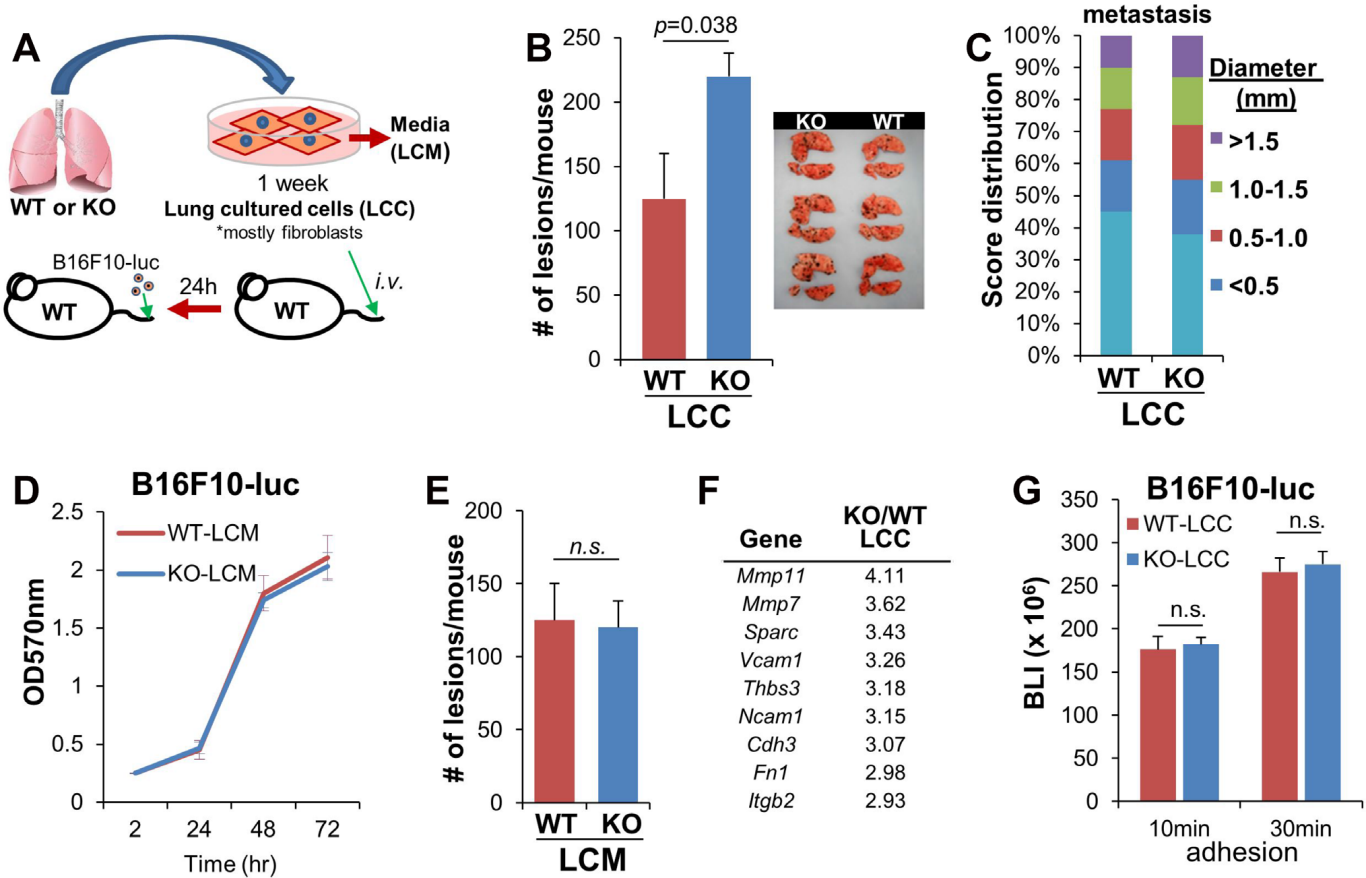
Loss of SSeCKS in lung fibroblasts enhances cancer cell colonization in the lung (**A**) Isolation and injection of lung fibroblasts. Single-cell cultures of WT or KO whole lungs for 1 week as “lung cultured cells” (LCC) resulted in the outgrowth of fibroblasts, which were injected *i.v.* into WT hosts, followed 1d later with the *i.v.* injection of B16F10-luc. (**B**) Number of the lung macrometastases after 14 d in WT mice pre-injected with WT- or KO-LCC. *n =* 6/group. Error bars represent SEM. (**C**) Percentage and size (diameter, in mm) of lung macrometastases of the mice injected in panel B, analyzed and quantified by Image J software. (**D**) Proliferation of B16F10-luc in media supplemented with 20% conditioned media from WT- or KO-LCC (LCM). Error bars, SEM of triplicate wells. (**E**) Number of lung macrometastases 14 d after co-injection of B16F10-luc cells resuspended in equal volumes of fresh WT- or KO-LCM (normalized for protein content) into WT hosts. *n =* 6/group; error bars, SEM; n.s., not significant. (**F**) Adhesion-regulating genes upregulated in KO- vs. WT-LCC. (**G**) Adhesion (for 10 or 30 min) by B16F10-luc on monolayers of WT- or KO-LCC, followed by 3 PBS washes and then luciferase assay on adherent cells. Error bars, SEM of two independent experiments done with triplicate wells; n.s., not significant.

We previously showed that the enhanced peritoneal metastasis induced by melanoma cells in KO (vs. WT) hosts was governed by increased chemokines secreted by membrane myofibroblasts [22]. To address whether lung KO fibroblasts directed the enhanced melanoma lung colonization described in Figure 1G, total KO and WT lungs from naïve mice were digested and plated as single-cell cultures (Figure 2A). CD102 staining on 1-week cultures indicated that most (>90%) of the “lung cultured cells” (LCC) cells were fibroblasts (data not shown). Co-injection (*i.v.*) of B16F10-luc with equal numbers of KO-LCC into WT hosts induced 1.75-fold more lung macrometastases than co-injection of WT-LCC (Figure 2B), although KO-LCC did not enhance orthotopic tumor formation over co-injection with WT-LCC (Figure 2C). In contrast to the ability of secreted stromal factors from KO mice to enhance peritoneal metastasis of melanoma cells [22], KO “lung conditioned media” (LCM), representing fresh media from the last 12h of the LCC culture, failed to either enhance B16F10-luc proliferation in 2D cultures (Figure 2D) or induce more lung metastases when mixed with *i.v.* injections of B16F10-luc in WT hosts (Figure 2E).

We then addressed whether LCC fibroblasts could facilitate the increased cell adhesion suggested by the enhanced melanoma colonization of KO host lungs in Figure 1G. A comparative transcriptome analysis indicated that several potential adhesion molecules were upregulated in KO- vs. WT LCC, including *Sparc, Vcam1, Tbs3, Cdh3, Fn1* and *Itgb2* (Figure 2F). Moreover, KO-LCC also upregulated two metalloproteinase genes, *Mmp11* and *Mmp7*. However, none of these factors is directly responsible for melanoma colonization in KO lungs because there was equal adhesion *in vitro* by B16F10-luc to KO- and WT-LCC monolayers (Figure 2G). Thus, the KO-LCC likely enhance metastatic colonization by attaching to and affecting endothelial cells.

**Figure 3:**
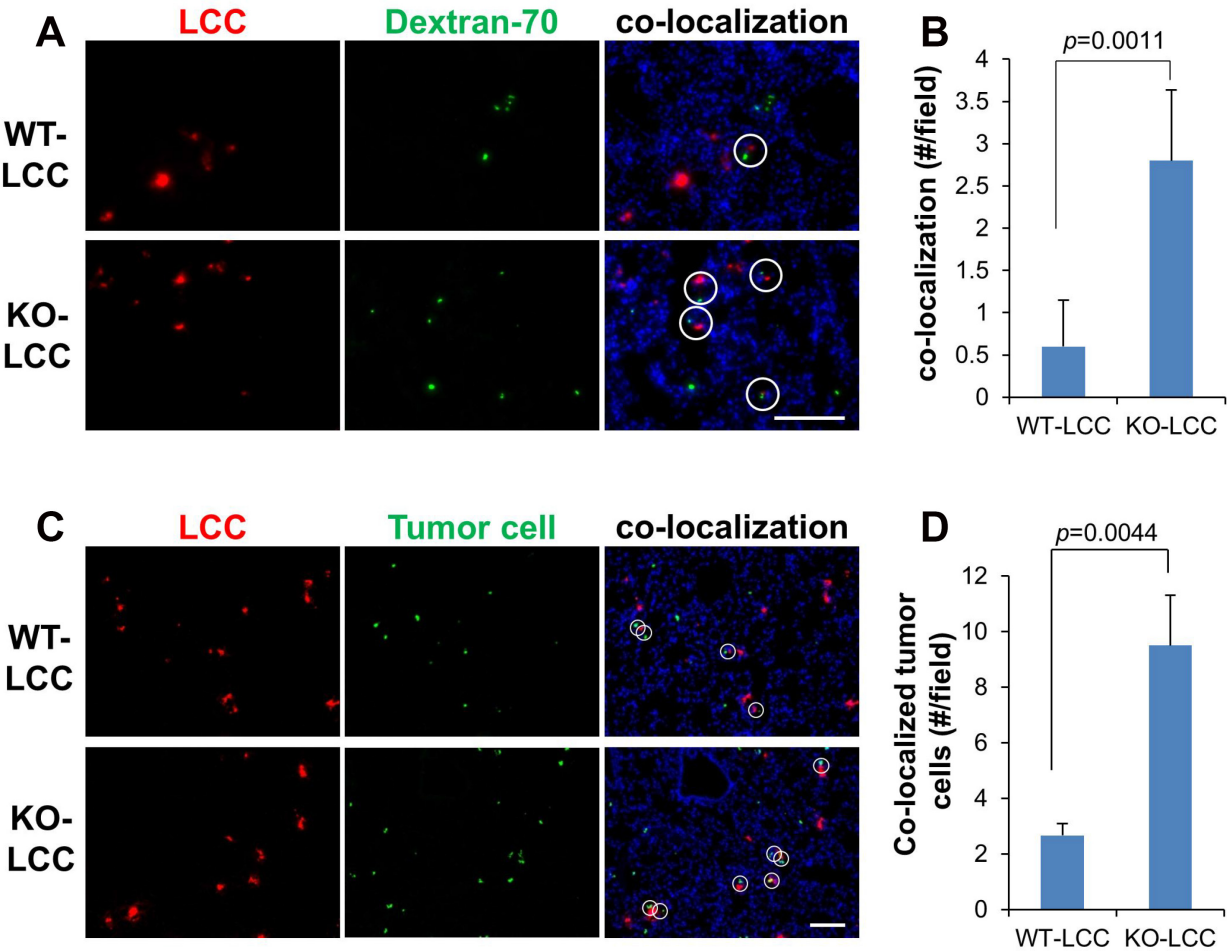
KO lung fibroblasts and melanoma cells home to foci of vascular leakiness in the lung Fluorescent lung images of the WT- or KO-LCC pre-labeled with Cell Tracker-Red 6 h after *i.v.* co-injection with FITC-dextran-70 (green) (**A**) or Cell Tracker-Green-labeled B16F10-luc (**C**), to monitor sites of vascular leakage. Nuclei (blue) were identified by DAPI staining. White circles, co-localization of fibroblasts with dextran-70 foci. Mean of fibroblast/dextran-70 (**B**) or fibroblast/tumor (**D**) co-localization per microscopic field. Error bars, SEM of 6 independent microscopic fields/lung for three lungs.

Hiratsuka *et al.* [10] showed that metastatic cells home to foci of lung vascular hyperpermeability, controlled by the FAK-dependent upregulation of E-Selectin in lung endothelial cells. Moreover, tumor-derived SPARC is known to promote metastatic colonization through increasing vascular permeability [27]. Because KO-LCC exhibit increased expression of *Sparc* and cell-cell adhesion protein genes (Figure 2F), we postulated that KO-LCC may enhance lung metastasis by enriching at sites of vascular leakage. KO- or WT-LCC labeled with Cell Tracker Red dye were co-injected *i.v.* into WT hosts with equal numbers of B16F10-luc cells plus FITC-labeled Dextran-70, as a marker of leaky vasculature [28]. After 6h, the hosts were sacrificed and their lungs analyzed for co-localization of LCC with leaky lung vessels, or in parallel, stained for tumor cells (using anti-luciferase plus FITC-labeled secondary Ab). Compared to levels with WT-LCC, there was increased co-localization with KO-LCC and both leaky vessels (Figure 3A and 3B) and tumor cells (Figure 3C and 3D), suggesting that KO-LCC modify lung endothelial cells to facilitate increased melanoma colonization through increased vascular leakiness.

**Figure 4:**
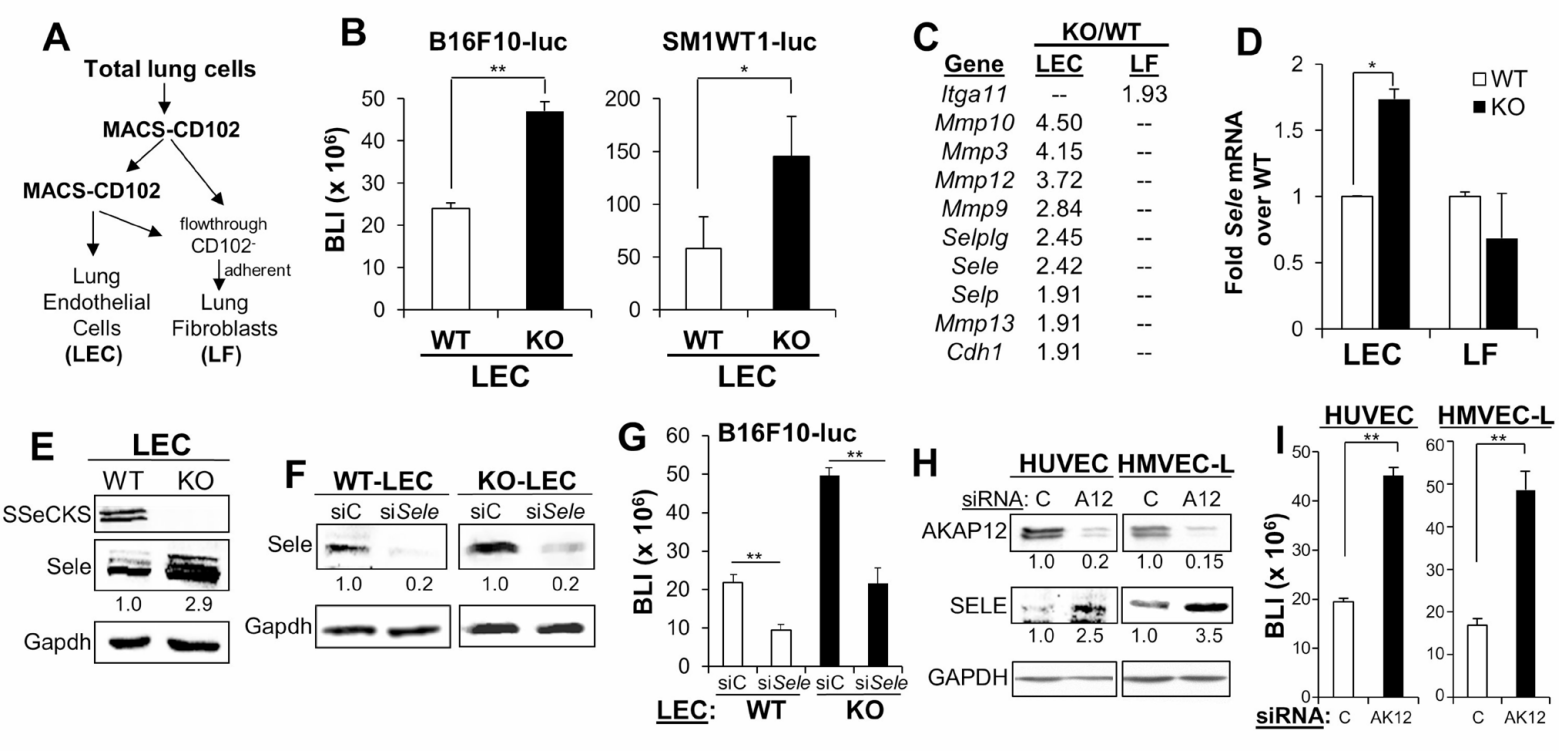
SSeCKS regulates the expression of E-Selectin and cancer cell adhesion on vascular endothelial cells (**A**) Protocol for the isolation of CD102^+^ lung endothelial cells (LEC) and CD102^-^ lung fibroblasts (LF). (**B**) BLI of adhered (10 min) B16F10-luc or SM1WT1-LM3-luc on WT- or KO-LEC. Error bars, SEM for triplicate wells with LEC from 6 WT or KO lungs tested independently. ^*^p < 0.01; ^**^p < 0.001. (**C**) PCR array analysis of adhesion-regulating genes upregulated in KO- vs. WT-LEC or LF. All *p* values are < 0.05. (**D**) Relative mRNA expression of E-Selectin (*Sele*) in WT- or KO-LEC and -LF, normalized to *Gapdh* levels. Error bars, SEM for triplicate assays on samples from 6 lungs. ^*^p < 0.01. (**E**) Immunoblot analysis of SSeCKS, Sele and Gapdh (protein loading control) from WT or KO-LEC. Band intensities were determined (for panels E, F and H) by Image J and normalized to Gapdh. (**F**) Immunoblot analysis of LEC treated (48 h) with control siRNA (siC) or siRNA for E-Selectin (si*Sele*). (**G**) Adhesion of B16F10-luc to WT- or KO-LEC treated with siC or *siSele.* Error bars, SEM of triplicate wells performed independently on LEC from 6 lungs. (**H**) Expression of AKAP12, SELE or GAPDH (as a protein loading control) in HUVEC and HMVEC-L pre-treated (48 h) with control siRNA (C) or siRNA to *AKAP12* (siA12) treatment. (**I**) Adhesion of B16F10-luc on HUVEC or HMVEC-L treated with siC or si*AKAP12* (siAK12) as in panel H. Error bars, SEM of triplicate wells from two independent experiments. ^**^p < 0.001.

In order to address whether the increased metastatic colonization in KO hosts could also be facilitated by an increase in the direct adhesion of melanoma cells to KO endothelial cells, CD102^+^ lung endothelial cells (LEC) were isolated by two rounds of MACS from single cell suspensions from naïve WT or KO lungs, resulting in >85% CD31-positive cells which readily formed vascular tubes *in vitro* (Supplementary Figure 2); lung fibroblasts (LF) were isolated as the CD102^-^ flow-through adherent cell fraction (Figure 4A), incapable of vascular tube formation (Supplementary Figure 2). We then assessed the efficiency of melanoma cells to adhere to monolayers of LEC, based on the luciferase reporter levels of the adherent cells remaining (after gentle washes) after 30 min. Both B16-F10-luc and SM1WT1-luc exhibited increased adherence to KO-LEC (Figure 4B and 4C). Microarray analysis identified increased expression of a number of adhesion molecule-expressing genes including *Selplg, Sele, Selp* and *Cdh1*, as well as increased expression of genes encoding Metalloproteinases 3, 9, 10, 12 and 13 in KO- vs. WT-LEC. In contrast, KO-LF only showed an increase in integrin_α_11 expression over WT-LF (Figure 4C). The increased E-Selectin RNA and protein levels in KO- vs. WT-LEC were confirmed independently (Figure 4D and 4E). Based on previous data showing the importance of surface E-Selectin for B16 colonization of the lung [15, 26], we treated WT- or KO-LEC with si*Sele*, confirmed E-Selectin knockdown (Figure 4F) and then performed the B16F10-luc endothelial monolayer adhesion assays described above. The loss of E-Selectin reduced melanoma adhesion to WT- or KO-LEC to the same extent, although adhesion to KO-LEC was relatively higher, as in Figure 4B (Figure 4G). Indeed, knockdown of *AKAP12*, the human orthologue of SSeCKS [19], in HUVEC and HMVEC-L (the latter, a closer kin to mouse LEC) induced E-Selectin expression 2.5- to 3.5-fold (Figure 4H), and enhanced adhesion to B16F10-luc (Figure 4I). Taken together, these data indicate that SSeCKS/*AKAP12* suppresses melanoma adhesion to endothelial cells based on reduction of E-Selectin expression.

**Figure 5:**
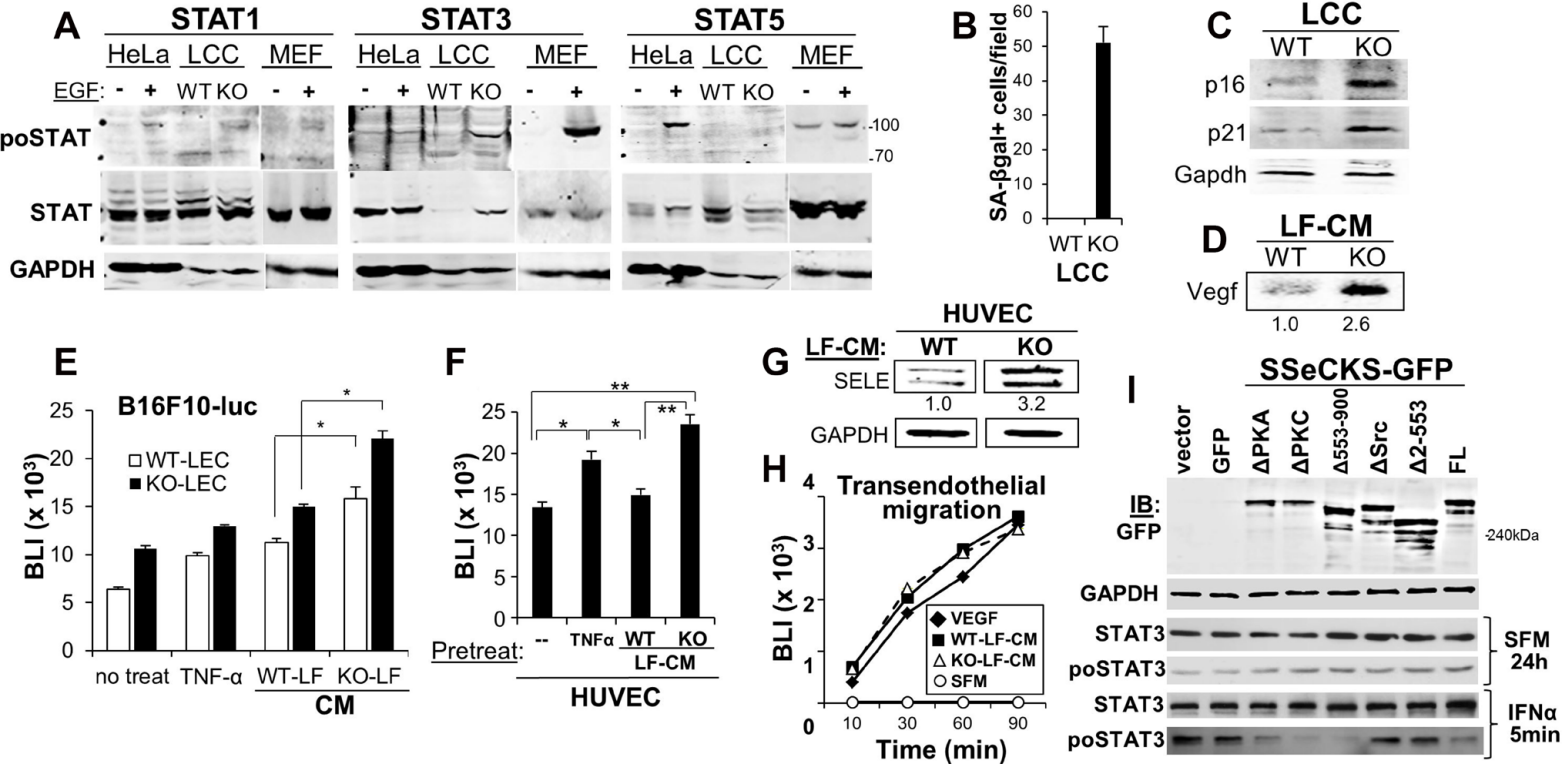
SSeCKS deficiency in lung fibroblasts results in premature senescence and SASP expression *in vitro* (**A**) Immunoblot analysis of relative Stat1, 3 or 5 phosphorylation (po- vs. total STAT) using lysates from hEGF-treated (100 ng/ml for 16 h) HeLa cells or mouse embryo fibroblast (MEF), or WT- or KO-LCC cells. GAPDH is assessed as a protein loading control. Protein molecular weight markers are shown at right. (**B**) Relative SA-βgal staining. Error bars, SEM from 6 independent microscopic fields, *p* < 0.001. Immunoblot analysis of WT- or KO-LCC lysates for p16, p21 or Gapdh (**C**) or LCM for secreted Vegf (**D**), quantified as described in Figure 4. (**E**) Adhesion of B16F10-luc on WT- or KO-LEC treated with serum-free media (SFM, “no treat”), TNF-α (16 h), or SFM containing 20% CM from WT- or KO-LF (2 h). Error bars, SEM of triplicate wells from three independent experiments. ^*^p < 0.01. (**F**) Same adhesion experiment as in panel E using HUVEC monolayers. Error bars, SEM of triplicate wells from three independent experiments. ^*^p < 0.01; ^**^p < 0.001. (**G**) Immunoblot analysis of SELE and GAPDH expression in HUVEC and HMVEC-L incubated for 2 h with CM (20% total) from WT- or KO-LF, imaged and normalized as described in Figure 4. (**H**) B16F10-luc migration through HUVEC monolayers towards SFM supplemented with VEGF or CM (20%) from WT- or KO-LF. Migrating cells (isolated from the bottom of Boyden chamber membranes) were assessed by luciferase assay. (**I**) Two days after transfection with pcDNA3.1/neo plasmids expressing GFP or SSeCKS-GFP fusions (FL, full-length; Δ, SSeCKS domain deletion), KO-LEC were incubated for 2 d in G418 (400 μg/ml)-containing media, incubated for 24 h in SFM, then treated for 5 min with IFNα. Lysates were then assessed by immunoblot for GFP, GAPDH, total STAT3 or STAT3^poY705^. A representative blot is shown for two independent experiments.

We sought to address the mechanism by which SSeCKS controls the ability of LF to “educate” LEC, thereby regulating adhesion of metastatic melanoma cells. Based on recent data showing that STAT3 activation in lung fibroblasts was critical to induce the secretion of senescence-associated pro-metastatic factors, including IL-6 and VEGF [29], we assessed the relative activation levels of STAT1, STAT3 and STAT5 in WT- or KO-LCC (highly enriched for fibroblasts), or in HeLa or mouse embryo fibroblasts, where EGF is known to induce activation of these STATs to varying degrees. Whereas KO-LCC showed a small STAT1 activation compared with levels in WT-LCC, STAT3 protein and activation levels were greatly induced in KO-LCC (Figure 5A). Indeed, KO-LCC exhibited highly increased levels of the senescence marker, SA-βgal (Figure 5B), consistent with our previous observation of increased premature senescence in KO-mouse embryo fibroblasts [30]. KO-LCC also showed increased levels of the senescence markers, p16 and p21 (Figure 5C), as well as Vegf (Figure 5D). To test whether these increased secreted senescence factors could “educate” LEC, monolayers of WT- or KO-LEC were incubated for 3h with CM from WT-or KO-LF (normalized for equal protein content), washed and then used for adherence assays by B16F10-luc cells. Although CM from WT-LF could enhance melanoma adhesion to both WT- and KO-LEC, KO-LF enhanced this adhesion about twofold more (Figure 5E). Addition of TNFα, known to enhance endothelial E-Selectin expression [31], was used as a positive control. Similar results were attained by treating HUVEC with CM from WT- or KO-LF, except that WT-LF-CM did not induce more B16F10-luc adhesion (Figure 5F). Consistent with this, incubation of HUVEC with CM from KO-LF induced a 3.2-fold increase in E-Selectin over levels induced by CM from WT-LF (Figure 5G). In contrast, CM from WT- or KO-LF were equally capable of inducing B16F10-luc migration through HUVEC monolayers (Figure 5H). Because this assay measures vascular leakiness of FITC-labeled dextran-70 solubilized in media containing LF-CM, or as a positive control, VEGF, this result suggests that secreted factors from KO-LF are only responsible for increased tumor cell adhesion to endothelial cells (due to increased E-Selectin expression).

Because SSeCKS controls multiple signaling pathways through its various scaffolding domains [19], we investigated which domains, and therefore, signaling pathways, were required for SSeCKS to suppress IFNα-induced STAT3 activation in KO-LF. Thus, KO-LF were transduced with vectors expressing GFP alone or SSeCKS-GFP constructs representing full-length (FL) or scaffolding domain-deleted mutants, as were described previously [32, 33, 22]. The Δ2-553 mutant is deleted of scaffolding domains for Src and phosphoinositol phosphates, whereas the Δ553-900 is deleted of scaffolding domains for PKC and PLK1 [22]. Of the SSeCKS constructs, only the Δ2-553 and ΔSrc mutants, the latter containing a more discrete deletion of the Src-binding domain between a.a. 152-166 [34], failed to suppress STAT3 activation (Figure 5I). Taken with the data above, these data indicate that increased endothelial E-Selectin can be induced by the increased secretion of LF factors that induce E-Selectin expression on endothelial cells *in trans*, and that the LF secretion is likely regulated by SSeCKS through its scaffolding of Src.

## DISCUSSION

Formation of the pre-metastatic niche is governed by a complex interplay between multiple cell types, including local endothelial cells, fibroblasts and myofibroblasts, as well as recruited immune and bone marrow-derived progenitor cells, all responding to factors secreted by distal tumor cells. Here, we analyze roles for the SSeCKS/AKAP12 metastasis suppressor in resident endothelial cells and fibroblasts that control metastatic colonization of melanoma cells in the lung, using syngeneic *Braf*^WT^ and *Braf*^C600E^ melanomas in immunocompetent WT or SSeCKS-null (KO) hosts. Our data show that the enhanced formation of lung metastases in KO hosts is governed at the level of initial colonization irrespective of *Braf* status, such that the increased colonization of KO lungs at 6h post-injection is similar to the increased level of melanoma cells remaining in KO vs. WT lungs 3 days later. Also, whereas spontaneous lung metastasis in WT hosts by B16F10 cells seems governed by tumor progression at the primary site (i.e.- metastases were identified in cases with the largest primary-site tumors), there was no bias to metastatic potential in KO hosts (i.e.- primary-site tumors of all sizes produced lung metastases). This is consistent with the KO lung being metastasis-prone.

The suggestion that KO lungs facilitate more melanoma adhesion is borne out by our finding that KO lung endothelial cells, but not KO lung fibroblasts, showed enhanced binding of melanoma cells *in vitro*, over WT controls. Increased binding of melanoma cells to HUVEC and HMVEC-L cells was similarly facilitated by *AKAP12* knockdown. AKAP12 deficiency in endothelial cells correlated with increased expression of E-Selectin, a known receptor for P-Selectin glycoprotein ligand-1 (Selplg/Psgl1) [15] and sialyl-Lewis-X/A ligands [26] on B16F10 cells. Importantly, E-Selectin knockdown abrogated the enhanced adhesion of B16F10 to KO-LEC, strongly suggesting that increased lung endothelial adhesion in this system is E-Selectin-dependent. Differential transcriptome analysis also identified P-Selectin and Cadherin-1 as adhesion proteins upregulated in KO- vs. WT-LEC, and interestingly, KO-LEC upregulated Selplg/Psgl-1 itself. Though the latter is typically found on the surface of various monocytes, lymphocytes, neutrophils and platelets, and facilitates rolling of these cells along the endothelium [35], its increased expression in KO-LEC may add to the increased adhesion/colonization of melanoma cells. Src signaling is required for the induction of E-Selectin in aortic endothelial cells [36], and although this pathway has not been confirmed in lung endothelial cells, it is likely that the loss of AKAP12 in our system correlates with increased Src-mediated E-Selectin expression, based on data that SSeCKS/AKAP12 can attenuate Src signaling without affecting intrinsic Src tyrosine kinase activity, by directly scaffolding Src away from integrin-FAK-growth factor receptor-rich membrane domains to lipid rafts [34].

The role of SSeCKS/AKAP12 in controlling crosstalk between LF and LEC is complex, yet our evidence suggest that it normally suppresses formation of the pre-metastatic niche by attenuating LF/LEC crosstalk. Although co-injection of KO-LCC (effectively equal to LF in that they were >90% fibroblasts) increased lung metastasis formation by B16F10 over WT-LCC co-injection controls, there was no increase in melanoma adhesion to KO-LCC, nor was the CM media from the KO-LCC more capable of inducing melanoma proliferation *in vitro* or metastasis formation *in vivo*. We attributed this to the increased ability of KO-LCC to home to foci of leaky lung vasculature, where they co-localize at increased frequency with injected melanoma cells. Sites of increased vascular permeability are known to foster increased metastatic colonization [4]. In our model, increased KO-LCC homing to these sites is likely facilitated by the upregulation of several adhesion or extracellular matrix proteins on KO-LCC, such as Vcam1, thrombospondin-3, Ncam1, cadherin-3, integrinβ2, and fibronectin1. The increased expression of MMPs by KO lung fibroblasts and endothelial cells may also contribute to increased vascular leakiness.

The loss of SSeCKS/Akap12 in LF increases the expression of senescence markers, such as SA-β-gal, p16, p21 and Vegf, concomitant with an increase in the activation of STAT3, and to a lesser extent, STAT1. STAT3 is a known mediator of the secretion of pro-metastatic factors by stroma in the pre-metastatic niche [37, 29], and moreover, increased metastatic angiogenesis promoted by the Src-dependent secretion of VEGF by these stroma through STAT3 [38]. Indeed, we show that CM from KO-LF enhances the adhesion of B16F10 cells to mouse LEC or HUVEC cells over levels induced by WT-LF-CM. Most significantly, CM from KO-LF is a much more potent inducer of E-Selectin expression in endothelial cells. Given that KO-LF-CM can induce melanoma adhesion to endothelial cells, but that co-injection (*i.v.*) of this CM with tumor cells has no enhancing effect, it is likely that the stromal enhancement is local, focused within pre-metastatic niches. Consistent with the notion that a Src-Stat3 axis in KO-LF drives melanoma adhesion on endothelial, we showed that re-expression of full-length SSeCKS or SSeCKS variants deleted of their PKA-, PKC- or PLK1-scaffolding domains, but not SSeCKS deleted of its Src-scaffolding domain, could suppress IFNα-induced STAT3 activation in KO-LF. It is important to note that we have not rules out the ability of KO-LF to directly affect tumor cells, specifically, their ability to adhere to endothelial cells, such has been reported previously [39].

There has been much focus recently on the interplay between secreted and adhesion factors induced through a complex crosstalk between tumor cells, local microenvironmental cells, immune cells and recruited bone-marrow stem cells that contribute to the formation of the pre-metastatic niche. However, there is still a dearth of knowledge regarding which pathways control these functions in specific cell populations. The current work underlines the role of stromal/endothelial crosstalk in regards to formation of the pre-metastatic lung niche that promotes the initial adhesion of melanoma cells. Our data are the first to focus on the important role played by scaffolding proteins such as SSeCKS/AKAP12 in controlling this process in stromal and endothelial cells through the scaffolding and signaling control of mediators such as Src.

## MATERIALS AND METHODS

### Cell line and culture

The B16-F10-luc-G5 (“B16F10-luc”) murine melanoma cell line stably expressing firefly luciferase was purchased from Perkin Elmer/Caliper Life Sciences (Akron, OH). SM1WT1-LM3-luc cells, exhibiting increased lung metastasis, were described previously [22]. These cells were maintained in RPMI1640 supplemented with 10% fetal bovine serum (FBS) and grown at 37° C in 5% CO_2_. HeLa cells were maintained in DMEM/10% FBS. WT and SSeCKS-null immortalized mouse embryonic fibroblasts (MEF) [30] were cultured in DMEM/10% FBS containing non-essential amino acids, L-glutamine, 2-mercaptoethanol, sodium pyruvate, HEPES and penicillin-streptomycin. Isolated murine lung endothelial cells (LEC) were maintained on gelatin-coated dishes in M199 medium supplemented with 15% FBS, Endothelial Cell Culture Supplement (BD Bioscience), heparin and glutamine. Human umbilical vein endothelial cells (HUVEC) and human lung microvascular endothelial cells (HMVEC-L) were cultured in EGM-2 medium (Lonza) supplemented with EGM-2 Single Quotes or EGM-2 MV Single Quotes (Lonza).

### Plasmid construction

Construction of SSeCKS-GFP in pcDNA3.1 was described previously [33] The following deletions were produced in pcDNA3.1/SSeCKS-GFP using long-run inverse PCR, as we described previously [40]: ΔSrc (a.a. 153-166) [34], ΔPKC (a.a. 596-605, 745-753) [32], ΔPKA (a.a. 1534-1553) [22], Δ553–900 [32] and Δ2-553 [33].

### Animal studies

All mouse care and experiments were performed in accordance with established institutional guidelines and approval by the Roswell Park Comprehensive Cancer Center Animal Care and Use Committee. SSeCKS/*Akap12*-null (KO) mice were generated as described previously [41]. C57BL/6J wild-type (WT) mice were obtained from Jackson Laboratory (Bar Harbor, ME, USA). Age-matched 5 to 7-week-old mice were used for cancer cell injection studies, whereas primary lung cells were isolated from 1- to 2-week-old mice.

### Experimental metastasis models

B16F10-luc (10^5^) or SM1WT1-LM3-luc (10^6^) cells were suspended in sterilized PBS and injected either *i.d.* or *i.v.* (tail vein) in WT or KO mice. 14 days later, mice were euthanized, perfused with sterile PBS followed by analysis of lung tissue. To measure bioluminescence (BLI), whole lungs were soaked in 15 mg/ml D-Luciferin solution (Caliper Life Sciences) for 10 min after dissection, and monitored by *in vivo* imaging (IVIS: Perkin Elmer/Caliper IVIS Spectrum; RPCI Translational Imaging Shared Resource, Mukund Seshadri, Director). For time course BLI measurements of lung tissue, post-euthanasia perfusion was performed with 10 ml PBS followed by freezing lungs in 1.5 ml tubes on dry ice after removal. Lysates of lung tissue were prepared with 500 µl lysis reagent provided from Luciferase Assay Systems (Promega), followed by centrifugation at 15,000 × g for 10 min to obtain cleared supernatants. Aliquots were subjected to firefly luciferase assays using Promega kits (Cat. #E1500) and BLI was measured using a Veritas luminometer (Turner Biosystems). Metastatic lesion sizes on surface of lungs were measured under the microscope and quantified with micro-scales, such that volumes (mm^3^) were determined by the formula, 0.52 × L × W × H.

### Fluorescence microscopy of lung

LF were labeled with 20 µM CMTPX (Molecular Probes) for 30 min at 37° C and injected *i.v.* into tail veins of WT mice. 24h later, FITC-conjugated dextran 70,000 MW (Sigma-Aldrich) or tumor cells stained with 20 µM CMFDA (Molecular Probes) were injected *i.v.* After 60 min for dextran and 6 h for tumor cells, mice were euthanized and perfused with 4% paraformaldehyde in PBS. Lungs were dissected and frozen in OCT solution, and sections were captured by a Nikon TE 2000-E inverted fluorescence microscope (Melville, NY, USA) using Metavue v.7.7.6.0 software (Molecular Devices, Sunnyvale, CA, USA). Positive cell numbers were quantified by manually counting, with at least 6 fields assessed per sample.

### Gene expression analyses

LCC gene expression was analyzed by the Rowell Park Genomics Shared Resource (Sean Glenn, Director) using Illumina MouseRef-8 v2.0 Expression BeadChips. Mouse LEC and LF matrix, adhesion and invasion genes were analyzed using a RT^2^ Profiler PCR Array (Qiagen; cat. 330522) according to the manufacturer’s instructions. RNA was isolated using Trizol (ThermoFisher Scientific).

### LEC and LF isolation-

Lungs were isolated from 1 to 2 week-old mice that were first perfused with sterilize PBS perfusion then with “lung dissociation solution” (Miltenyi Biotec, Germany) administered through the trachea, minced and incubated an additional 30 min at 37° C with lung dissociation solution. After passing through sterile 40 μm cell strainers and then shaking vigorously for 15 sec, single-cell suspensions were seeded in M199 medium supplemented with 15% FBS, Endothelial Cell Culture Supplement, heparin and glutamine on gelatin-coated dishes. Sub-confluent cultures were trypsinized and then passed through MACS (magnet activated cell sorting) anti-Rat IgG kappa magnetic beads (Miltenyi Biotec, Germany) coated with CD102 antibody (BioLegend, Inc.). After three PBS washes while on the magnet, cells were eluted in PBS off the magnet, and then seeded at 10^6^ per gelatin-coated 60 mm dish. After 4–5 d incubation, the cells were trypsinized and subjected to another round of MACS sorting to increase endothelial purity (>90%). Lung fibroblasts (LF) were isolated as the CD102^-^ flow through cells that quickly adhered to dishes and that grew in DMEM/10% FBS, non-essential amino acids, glutamine, 2-mercaptoethanol, sodium pyruvate, HEPES and penicillin-streptomycin. After proliferating for 7 d, these cells exhibited >90% vimentin-positivity.

### Tumor adhesion assay

WT- or KO-LEC or –LF, or HUVEC or HMVEC-L, were seeded onto 24-well plates and cultured until confluency. B16F10-luc or SM1WT1-luc cells in RPMI-1640/10% FBS were seeded at 2.5 × 10^3^ cells/well and allowed to adhere for 10–30 min. Unattached cells were removed with three gentle PBS washes. Attached cells were quantified via BLI using the Dual-Glo Luciferase assay according to manufacturer’s procedure (Promega). To assess conditioned media (CM) effects, endothelial monolayers were incubated for 3 h with either 50 ng of TNF-α (positive control) or fresh LF-CM (cultured 16 h in serum-free DMEM) prepared as a 20% final volume. Melanoma adherence was measured as above.

### Transendothelial migration assay

HUVEC were seeded atop Boyden chamber inserts (8 μm pores) coated with gelatin and cultured to confluency. FITC-conjugated dextran 70,000 MW (Sigma-Aldrich) dissolved in either serum-free DMEM or conditioned medium from WT- or KO-LF, was placed atop the endothelial monolayer, with serum-free DMEM or conditioned medium from LF placed in the culture wells. 50 µl of medium from the culture well was collected at various time points (10–90 min), diluted into 950 µl serum-free DMEM and fluorescence measured at emission/excitation 485/530 nm using a SpectraMax M2 multi-plate reader (Molecular Device).

### *In vitro* proliferation assays

B16F10 tumor cells were seeded 2 × 10^3^ cells/well in 96-well culture plates. After 2, 24, 48, 72 and 96 h, the medium was removed and the cells fixed with cold methanol for 10 min. The cells were then treated with 100 µl of 0.5% crystal violet solution (50 mg crystal violet in 25% methanol) 10 min, followed by extensive washing with water and complete drying. Stained cells were dissolved for 20 min in 100 µl of 10% acetic acid, and then absorption was measured at 595 nm using a microplate reader (SpectraMax-M2, Molecular Devices, Sunnyvale, CA, USA).

### SA-βgal staining

Staining was performed as described previously [30].

### Immunoblotting (IB)

Cells were lysed RIPA buffer [42] containing 1 mM each of phenylmethanesulfonylfluoride, Na_3_VO_4_, NaF plus one Complete Protease Inhibitor (Roche, Indianapolis, IN, USA) tablet per 10 ml. Lysates were cleared by centrifugation at 15,000 × g for 10 min after sonication. The following antibodies were used: CXCR3 (1:500, sc-13951, Santa Cruz, Santa Cruz, CA, USA), p16 (1:500, sc-1661, Santa Cruz), p21 (1:500, sc-397, Santa Cruz), Stat1 (1:1000, sc-346, Santa Cruz), Stat3 (1:1000, sc-482, Santa Cruz), Stat5 (1:1000, #9363, Cell Signaling, Danvers, MA, USA), po-Stat1 (1:1000, #9171, Cell Signaling), po-Stat3 (1:1000, sc-8059, Santa Cruz), po-Stat5 (1:1000, #9351, Cell Signaling) and Gapdh (1:1000, sc-25778, Santa Cruz). For the analysis of soluble protein expression profiles of the PF, we used the Proteome Profiler Array mouse chemokine array kit (R&D Systems) using 200 µl of WT- or KO-PF. The intensities of the immunoreactive IB bands and array spots were quantified by densitometry using ImageJ software (NIH).

### RT-PCR and quantitative real-time PCR

Total RNA fraction was extracted from cells using the TRIZOL reagent (ThermoFisher-Invitrogen, Grand Island, NY, USA), and was reverse transcribed using Superscript III reverse transcriptase (Invitrogen) for RT-PCR and High capacity cDNA reverse transcription kit (ThermoFisher-Applied Biosystems, Grand Island, NY, USA) for quantitative real-time PCR. Quantitative real-time PCR was performed on a 7900HT real-time PCR system (Applied Biosystems) using SYBR Green PCR Core Reagents (Applied Biosystems). The relative expression was normalized to β-actin.

### Statistical analyses

Data were expressed as mean ± SEM, with all experiments repeated independently at least twice. Differences were analyzed by one-way ANOVA or Student’s *t* test. *P* < 0.05 was considered significant.

## SUPPLEMENTARY MATERIALS FIGURES



## Abbreviations

AKAP12L: A kinase anchoring protein 12
ANOVA: Analysis of variance
BLI: Bioluminescence Imaging/Index
CM: conditioned media
EGF: epidermal growth factor
FBS: fetal bovine serum
FITC: fluorescein isothiocyanate
FL: full length
GFP: green fluorescent protein
HUVEC: human umbilical vascular endothelial cells
HMVEC-L: human microvascular lung endothelial cells
IFN: interferon
*i.d.*: intradermal
*i.v.*: intravenous
KO: knockout
LCC: lung cultured cells
LCM: lung conditioned media
LEC: lung endothelial cells
LF: lung fibroblasts
LM3: lung metastasis 3
Luc: luciferase
MACS: magnetic-activated cell sorting
MEF: mouse embryo fibroblast
NS: not significant
PIP: phosphoinositol phosphates
PKA: protein kinase A
PKC: protein kinase C
PLK-1: polo-like kinase-1
SEM: standard error of the mean
SFK: Src-family kinases
SSeCKS: Src Suppressed C Kinase Substrate
STAT: signal transducer and activator of transcription
RT-PCR: reverse transcriptase-polymerase chain reaction
TME: tumor microenvironment
TNF: tumor necrosis factor
VEGF: vascular endothelial growth factor
WT: wild-type

## References

[1] Fidler IJ, Kripke ML (2015). The challenge of targeting metastasis. Cancer Metastasis Rev.

[2] Sceneay J, Smyth MJ, Möller A (2013). The pre-metastatic niche: finding common ground. Cancer Metastasis Rev.

[3] Liu Y, Cao X (2016). Characteristics and Significance of the Pre-metastatic Niche. Cancer Cell.

[4] Peinado H, Zhang H, Matei IR, Costa-Silva B, Hoshino A, Rodrigues G, Psaila B, Kaplan RN, Bromberg JF, Kang Y, Bissell MJ, Cox TR, Giaccia AJ (2017). Pre-metastatic niches: organ-specific homes for metastases. Nat Rev Cancer.

[5] Hiratsuka S, Watanabe A, Aburatani H, Maru Y (2006). Tumour-mediated upregulation of chemoattractants and recruitment of myeloid cells predetermines lung metastasis. Nat Cell Biol.

[6] Becker A, Thakur BK, Weiss JM, Kim HS, Peinado H, Lyden D (2016). Extracellular Vesicles in Cancer: Cell-to-Cell Mediators of Metastasis. Cancer Cell.

[7] Kaplan RN, Riba RD, Zacharoulis S, Bramley AH, Vincent L, Costa C, MacDonald DD, Jin DK, Shido K, Kerns SA, Zhu Z, Hicklin D, Wu Y (2005). VEGFR1-positive haematopoietic bone marrow progenitors initiate the pre-metastatic niche. Nature.

[8] Yoshimura T, Howard OM, Ito T, Kuwabara M, Matsukawa A, Chen K, Liu Y, Liu M, Oppenheim JJ, Wang JM (2013). Monocyte chemoattractant protein-1/CCL2 produced by stromal cells promotes lung metastasis of 4T1 murine breast cancer cells. PLoS One.

[9] Läubli H, Borsig L (2010). Selectins promote tumor metastasis. Semin Cancer Biol.

[10] Hiratsuka S, Goel S, Kamoun WS, Maru Y, Fukumura D, Duda DG, Jain RK (2011). Endothelial focal adhesion kinase mediates cancer cell homing to discrete regions of the lungs via E-selectin up-regulation. Proc Natl Acad Sci USA.

[11] St Hill CA (2011). Interactions between endothelial selectins and cancer cells regulate metastasis. Front Biosci.

[12] Yamada M, Yanaba K, Hasegawa M, Matsushita Y, Horikawa M, Komura K, Matsushita T, Kawasuji A, Fujita T, Takehara K, Steeber DA, Tedder TF, Sato S (2006). Regulation of local and metastatic host-mediated anti-tumour mechanisms by L-selectin and intercellular adhesion molecule-1. Clin Exp Immunol.

[13] Sharma R, Sharma R, Khaket TP, Dutta C, Chakraborty B, Mukherjee TK (2017). Breast cancer metastasis: putative therapeutic role of vascular cell adhesion molecule-1. Cell Oncol (Dordr).

[14] Ohyama C, Tsuboi S, Fukuda M (1999). Dual roles of sialyl Lewis X oligosaccharides in tumor metastasis and rejection by natural killer cells. EMBO J.

[15] Yamaoka T, Fujimoto M, Ogawa F, Yoshizaki A, Bae SJ, Muroi E, Komura K, Iwata Y, Akiyama Y, Yanaba K, Shimizu K, Sato S (2011). The roles of P- and E-selectins and P-selectin glycoprotein ligand-1 in primary and metastatic mouse melanomas. J Dermatol Sci.

[16] Yu H, Lee H, Herrmann A, Buettner R, Jove R (2014). Revisiting STAT3 signalling in cancer: new and unexpected biological functions. Nat Rev Cancer.

[17] Kim KJ, Kwon SH, Yun JH, Jeong HS, Kim HR, Lee EH, Ye SK, Cho CH (2017). STAT3 activation in endothelial cells is important for tumor metastasis via increased cell adhesion molecule expression. Oncogene.

[18] Krtolica A, Parrinello S, Lockett S, Desprez PY, Campisi J (2001). Senescent fibroblasts promote epithelial cell growth and tumorigenesis: a link between cancer and aging. Proc Natl Acad Sci USA.

[19] Gelman IH (2012). Suppression of tumor and metastasis progression through the scaffolding functions of SSeCKS/Gravin/AKAP12. Cancer Metastasis Rev.

[20] Ko HK, Akakura S, Peresie J, Goodrich DW, Foster BA, Gelman IH (2014). A transgenic mouse model for early prostate metastasis to lymph nodes. Cancer Res.

[21] Su B, Zheng Q, Vaughan MM, Bu Y, Gelman IH (2006). SSeCKS metastasis-suppressing activity in MatLyLu prostate cancer cells correlates with VEGF inhibition. Cancer Res.

[22] Muramatsu M, Gao L, Peresie J, Balderman B, Akakura S, Gelman IH (2017). SSeCKS/AKAP12 scaffolding functions suppress B16F10-induced peritoneal metastasis by attenuating CXCL9/10 secretion by resident fibroblasts. Oncotarget.

[23] Akakura S, Bouchard R, Bshara W, Morrison C, Gelman IH (2011). Carcinogen-induced squamous papillomas and oncogenic progression in the absence of the SSeCKS/AKAP12 metastasis suppressor correlate with FAK upregulation. Int J Cancer.

[24] McLean GW, Komiyama NH, Serrels B, Asano H, Reynolds L, Conti F, Hodivala-Dilke K, Metzger D, Chambon P, Grant SG, Frame MC (2004). Specific deletion of focal adhesion kinase suppresses tumor formation and blocks malignant progression. Genes Dev.

[25] McLean GW, Brown K, Arbuckle MI, Wyke AW, Pikkarainen T, Ruoslahti E, Frame MC (2001). Decreased focal adhesion kinase suppresses papilloma formation during experimental mouse skin carcinogenesis. Cancer Res.

[26] Zhang J, Nakayama J, Ohyama C, Suzuki M, Suzuki A, Fukuda M, Fukuda MN (2002). Sialyl Lewis X-dependent lung colonization of B16 melanoma cells through a selectin-like endothelial receptor distinct from E- or P-selectin. Cancer Res.

[27] Tichet M, Prod’Homme V, Fenouille N, Ambrosetti D, Mallavialle A, Cerezo M, Ohanna M, Audebert S, Rocchi S, Giacchero D, Boukari F, Allegra M, Chambard JC (2015). Tumour-derived SPARC drives vascular permeability and extravasation through endothelial VCAM1 signalling to promote metastasis. Nat Commun.

[28] McNaught EI, Foulds WS, Johnson NF (1981). The permeability of the posterior blood ocular barrier after xenon photocoagulation: a study using fluorescein labelled dextrans. Br J Ophthalmol.

[29] Wang L, Cao L, Wang H, Liu B, Zhang Q, Meng Z, Wu X, Zhou Q, Xu K (2017). Cancer-associated fibroblasts enhance metastatic potential of lung cancer cells through IL-6/STAT3 signaling pathway. Oncotarget.

[30] Akakura S, Nochajski P, Gao L, Sotomayor P, Matsui S, Gelman IH (2010). Rb-dependent cellular senescence, multinucleation and susceptibility to oncogenic transformation through PKC scaffolding by SSeCKS/AKAP12. Cell Cycle.

[31] Ritchie AJ, Johnson DR, Ewenstein BM, Pober JS (1991). Tumor necrosis factor induction of endothelial cell surface antigens is independent of protein kinase C activation or inactivation. Studies with phorbol myristate acetate and staurosporine. J Immunol.

[32] Guo LW, Gao L, Rothschild J, Su B, Gelman IH (2011). Control of protein kinase C activity, phorbol ester-induced cytoskeletal remodeling, and cell survival signals by the scaffolding protein SSeCKS/GRAVIN/AKAP12. J Biol Chem.

[33] Ko HK, Guo LW, Su B, Gao L, Gelman IH (2014). Suppression of chemotaxis by SSeCKS via scaffolding of phosphoinositol phosphates and the recruitment of the Cdc42 GEF, Frabin, to the leading edge. PLoS One.

[34] Su B, Gao L, Meng F, Guo LW, Rothschild J, Gelman IH (2013). Adhesion-mediated cytoskeletal remodeling is controlled by the direct scaffolding of Src from FAK complexes to lipid rafts by SSeCKS/AKAP12. Oncogene.

[35] Kappelmayer J, Nagy B (2017). The Interaction of Selectins and PSGL-1 as a Key Component in Thrombus Formation and Cancer Progression. BioMed Res Int.

[36] Alamanda V, Singh S, Lawrence NJ, Chellappan SP (2012). Nicotine-mediated induction of E-selectin in aortic endothelial cells requires Src kinase and E2F1 transcriptional activity. Biochem Biophys Res Commun.

[37] Chang Q, Bournazou E, Sansone P, Berishaj M, Gao SP, Daly L, Wels J, Theilen T, Granitto S, Zhang X, Cotari J, Alpaugh ML, de Stanchina E (2013). The IL-6/JAK/Stat3 feed-forward loop drives tumorigenesis and metastasis. Neoplasia.

[38] Gray MJ, Zhang J, Ellis LM, Semenza GL, Evans DB, Watowich SS, Gallick GE (2005). HIF-1alpha, STAT3, CBP/p300 and Ref-1/APE are components of a transcriptional complex that regulates Src-dependent hypoxia-induced expression of VEGF in pancreatic and prostate carcinomas. Oncogene.

[39] Liu G, Feng S, Jia L, Wang C, Fu Y, Luo Y (2018). Lung fibroblasts promote metastatic colonization through upregulation of stearoyl-CoA desaturase 1 in tumor cells. Oncogene.

[40] Bu Y, Gelman IH (2007). v-Src-mediated down-regulation of SSeCKS metastasis suppressor gene promoter by the recruitment of HDAC1 into a USF1-Sp1-Sp3 complex. J Biol Chem.

[41] Akakura S, Huang C, Nelson PJ, Foster B, Gelman IH (2008). Loss of the SSeCKS/Gravin/AKAP12 gene results in prostatic hyperplasia. Cancer Res.

[42] Lin X, Tombler E, Nelson PJ, Ross M, Gelman IH (1996). A novel src- and ras-suppressed protein kinase C substrate associated with cytoskeletal architecture. J Biol Chem.

