# Supplementary material for: SSeCKS/Akap12 suppresses metastatic melanoma lung colonization by attenuating Src-mediated pre-metastatic niche crosstalk
Source: Oncotarget. 2018 Sep 11;9(71):33515–27. doi: 10.18632/oncotarget.26067 (PMC6173366; doi:10.18632/oncotarget.26067)
Supplement: Supplementary file 1 [file oncotarget-09-33515-s001.pdf]

SSeCKS/Akap12 suppresses metastatic melanoma lung colonization by attenuating Src-mediated pre-metastatic niche crosstalk

SUPPLEMENTARY MATERIALS

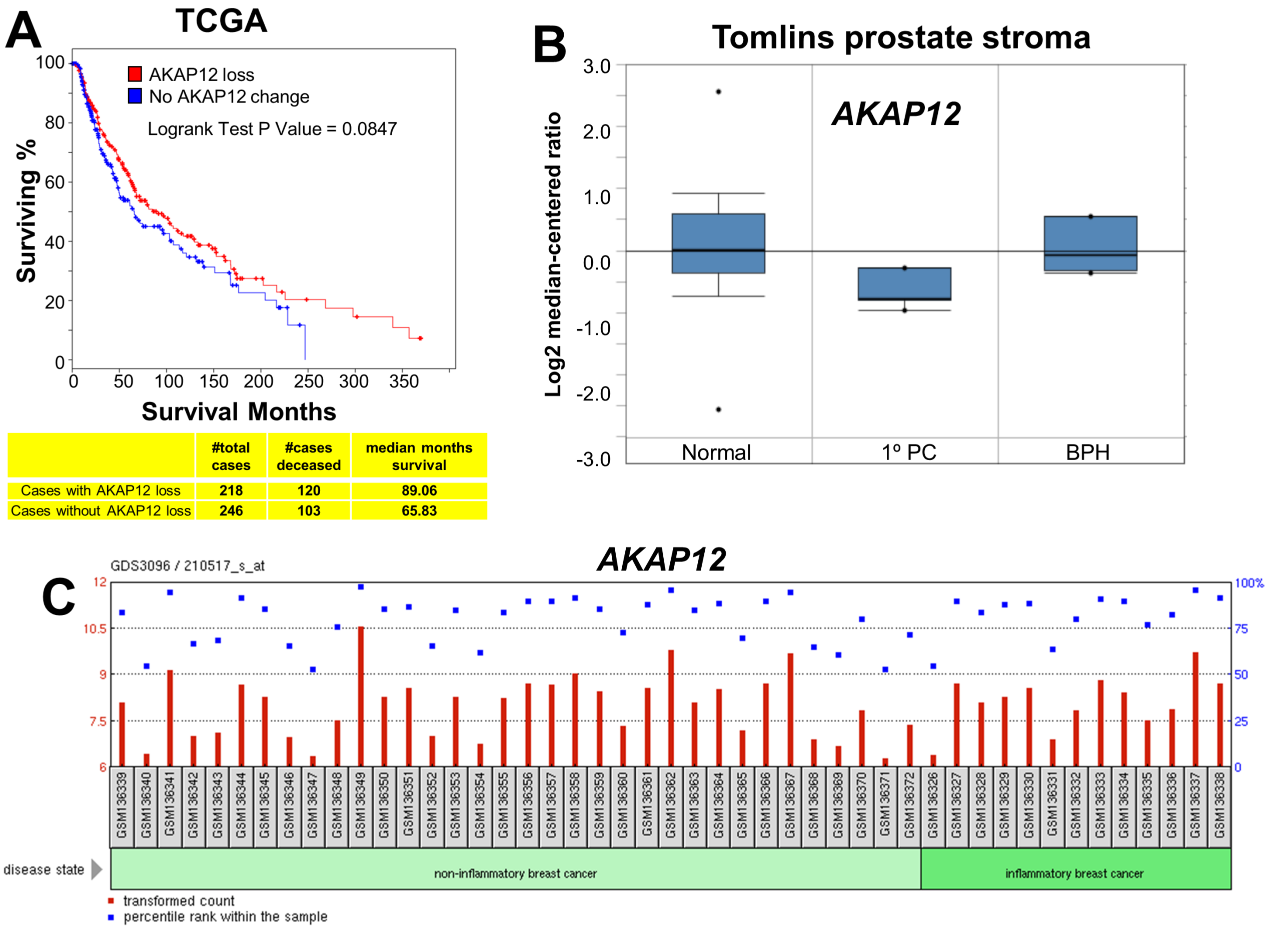

Supplementary Figure 1: AKAP12 expression in melanoma survival, or in human prostate- or breast-associated stroma. (**A**) Correlation of relative *AKAP12* expression with survival in the TCGA cohort (total $n = 464$), the p value being not significant. (**B**) Relative *AKAP12* expression of normal, primary tumor (1ºPC) and benign prostatic hyperplasia (BPH)-associated stroma in human prostate cancers from the Oncomine Tomlins Prostate dataset. (**C**) Relative *AKAP12* expression in the stroma from inflammatory vs. non-inflammatory human breast cancers from GEO study GDS3096.

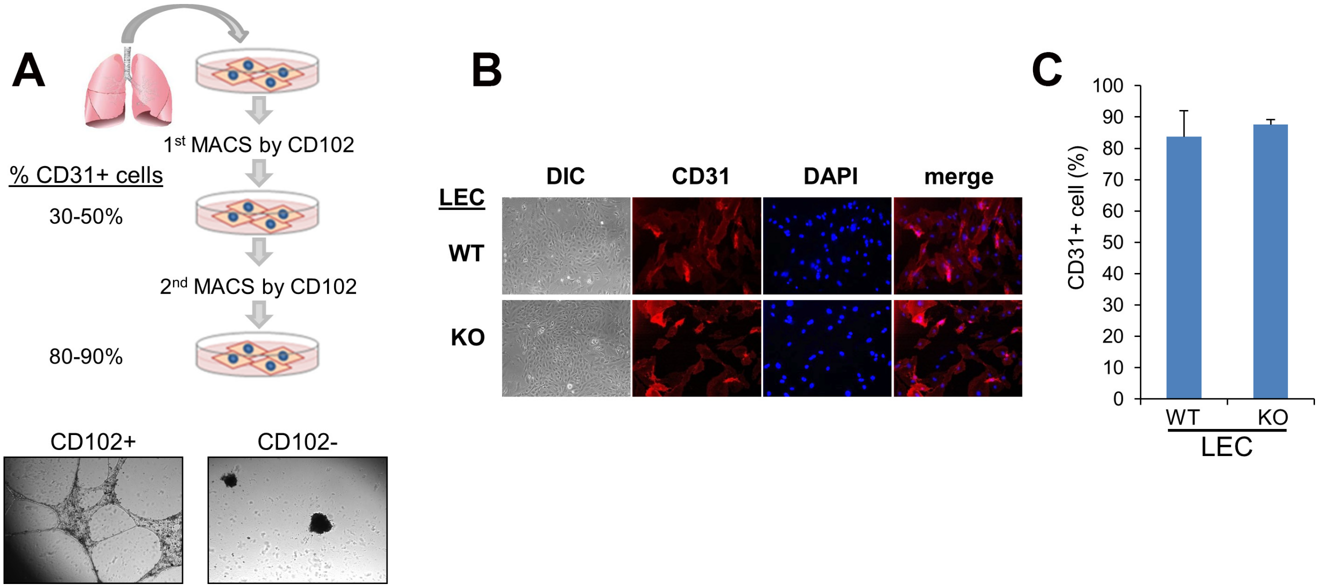

Supplementary Figure 2: Confirmation of endothelial enrichment after CD102 MACS. (**A**) *Top*. FACS quantification of CD31 positivity after 1 of 2 rounds of MACS-CD102 selection of mouse lung cells. *Bottom*. Functional confirmation of LEC after second MACS-CD102 round using a tube formation assay [21]. Note that the CD102^{-} flow-through fraction, which represented LF, failed to form tubes, whereas the CD102^{+} LEC did. (**B**) CD31 and DAPI staining of WT- and KO-LEC after the 2nd MACS-CD102 selection. (**C**) Percentage of CD31^{+} cells assessed by FACS analysis after two rounds of MACS-CD102 selection. Error bars, SEM of LEC from 6 lungs.
